# The gut microbiome as mediator between diet and its impact on immune function

**DOI:** 10.1038/s41598-022-08544-y

**Published:** 2022-03-25

**Authors:** Huiqing Shi, Rob ter Horst, Suzanne Nielen, Mirjam Bloemendaal, Martin Jaeger, Irma Joosten, Hans Koenen, Leo A. B. Joosten, Lizanne J. S. Schweren, Alejandro Arias Vasquez, Mihai G. Netea, Jan Buitelaar

**Affiliations:** 1 Department of Cognitive Neuroscience, Donders Institute for Brain, Cognition and Behaviour, Radboud University Nijmegen Medical Centre, Radboudumc, Kapittelweg 29, 6525 EN Nijmegen, Gelderland The Netherlands; 2 Department of Internal Medicine, Radboudumc Center for Infectious Diseases, Radboudumc, 6500 HB Nijmegen, Gelderland The Netherlands; 3 Department of Laboratory Medicine, Radboudumc, 6500 HB Nijmegen, Gelderland The Netherlands; 4 Interdisciplinary Center Psychopathology and Emotion Regulation, University Medical Center Groningen, University of Groningen, 9700 RB Hanzeplein 1, Groningen, The Netherlands; 5 Department of Immunology and Metabolism, Life and Medical Sciences Institute (LIMES), University of Bonn, Bonn, Germany; 6 Karakter Child and Adolescent Psychiatry University Centre, 6525 GC Nijmegen, Gelderland The Netherlands; 7 Department of Psychiatry, and Department of Genetics, Donders Institute for Brain, Cognition and Behaviour, Radboudumc, 6525 EN Nijmegen, Gelderland The Netherlands

**Keywords:** Medical research, Risk factors

## Abstract

Dietary habits may affect inflammatory status in humans. Here we explore this interaction as well as the potential mediating role of the gut microbiome (GM), given that the GM is both involved in processing of dietary components and influences the immune system. A cross-sectional analysis of a sample of 482 healthy participants (207 males and 275 females) was performed. Dietary intake was assessed by a semiquantitative food questionnaire. Adipokines and soluble inflammatory mediators were assayed with multiple immunoassays and ELISA. Microbial DNA was extracted from frozen stool samples of 471 participants. Polychoric correlation analysis was used to establish dietary patterns, and joint multivariate associations between these dietary patterns and immune biomarkers were studied using regression analyses with adjustment for sex, age, BMI, smoking, education levels and physical exercise and other dietary patterns. Non-parametric entropy mediation was applied to investigate whether diet-immune relationships are mediated by abundance of microbial species. In this cohort, we identified three dietary patterns, characterized as “high-meat” (meat and sweetened drink), “prudent diet” (fish, fruit, legumes and vegetables) and “high alcohol” (higher alcohol consumption). Higher adherence to prudent diet was associated with a higher adiponectin level. The high alcohol pattern was associated with high concentrations of circulating concentrations of pro-inflammatory markers (CRP, IL-6, VEGF). *Dialister invisus* was found to mediate the relationship between a prudent dietary pattern and adiponectin, AAT, CRP, IL-6, and VEGF. In conclusion, a meat-based diet and a diet with high alcohol consumption were associated with high concentrations of biomarkers of chronic low-grade inflammation, and conversely, a prudent diet was associated with anti-inflammatory biomarkers. Diet-inflammation regulation may differ between sexes. Mediation analyses revealed that the association between prudent diet and immune function was partially mediated by the GM. The study adds to our understanding of the associations between diet, the immune system and the GM in a healthy population.

## Introduction

Diet has a substantial influence on multiple facets of human health, and an unhealthy dietary pattern (e.g. high-fat Western-type diet) has been linked to increased risk for an array of chronic disorders such as atherosclerosis, metabolic syndrome, and obesity^1^. The immune system is emerging as a key intermediary in this relationship through food-induced modulation of pro-/anti-inflammatory factors that have been associated with chronic inflammatory conditions^2^ and by increasing/reducing risks for various pathological outcomes^3^.

In support of this notion are large epidemiological studies which demonstrated that a dietary pattern characterized by high intake of saturated fat and low fiber is associated with elevated levels of pro-inflammation biomarkers such as C-reactive protein (CRP) and interleukin (IL)-6^4^. Conversely, a dietary pattern with high intake of fruit and vegetables and/or regular fish consumption is associated with higher serum concentrations of adiponectin, which has anti-inflammatory properties^5^. These observational studies were further supported by interventional trials, which showed that diet may affect the serum inflammatory biomarker profiles. For example, a dietary intervention with high-cholesterol food increased CRP and serum amyloid A concentrations in insulin-sensitive participants^6^. Nevertheless, other studies into the immune modulatory effect of diet have produced inconsistent results^7^.

The gut microbiome (GM) helps metabolize nutrients, complementing the host’s metabolism^8,9^. Not only does the GM help extract nutrients, the microbiota also help calibrate immune responses in such a way that the GM and immune system constantly interact to create a gut-immune homeostasis^10^. One example is the microbial production of short-chain fatty acids (SCFAs) that possess anti-inflammatory properties and modulate cytokine production^11^. The interaction between diet, the GM and the immune system occurs though several mechanisms, for instance through dietary-derived microbial metabolites, diet-induced alteration of GM composition, alteration of the fitness of immunomodulatory microbes and by modulations in host metabolism^12^. Identifying the mechanisms of diet-immune interactions could help us regulate the effect diet has on the immune system through modulation of the GM. Because the GM can be manipulated easily using antibiotics and probiotics, the microbial community is a particularly suitable target for modulation^13^.

The study presented here was designed to (1) characterize the association between dietary patterns and immune characteristics in the 500FG study and (2) investigate whether the GM plays a mediating role in this relationship.

## Results

### Characteristics of the study population

The study population consisted of 207 men and 275 women with a mean age of 28.7 and 26.5 years, respectively (Table 1). Men had a higher average BMI than did women (23.3 versus 22.2 kg/m^2^, *P* < 0.001). 17.4% of the men and 9.8% of the women were currently smoking (*P* = 0.035). Men and women had similar levels of physical activity (*P* = 0.060) and educational attainment (*P* = 0.152).

**Table 1:** Characteristics of study subjects by sex.

Characteristics	Males (n = 207)	Females (n = 275)	*P*^a^ value
Age (y)	28.7 ± 13.6^b^	26.5 ± 11.5	0.059
Body mass index (kg/m^2^)	23.3 ± 2.8	22.2 ± 2.6	< 0.001
**Smoking**			
Current smoker	36 (17.4)^b^	27 (9.8)	0.035
Less than 10 cigarettes/d	23 (63.9)	19 (70.4)	
10 and more cigarettes/d	13 (36.1)	8 (29.6)	
Former smoker	33 (15.9)	33 (12.0)	
Never smoker	122 (58.9)	189 (68.7)	
Passive smoker	15 (7.2)	24 (8.7)	
**Physical activity level**			
Less than twice/week	66 (31.9)	99 (36.0)	0.060
2–4 times/week	90 (43.5)	133 (48.4)	
5 times or more/week	50 (24.2)	43 (15.6)	
**Educational attainment**			
Below college	30 (14.5)	27 (9.8)	0.152
College or current college training	177 (85.5)	248 (90.2)	

^a^*P* values for sex differences are based on t tests for continuous variables and chi-square tests for categorical variables.

^b^Values are mean ± standard deviation for continuous variables and number (percentage) for categorical variables.

### Dietary patterns

**Figure 1:**
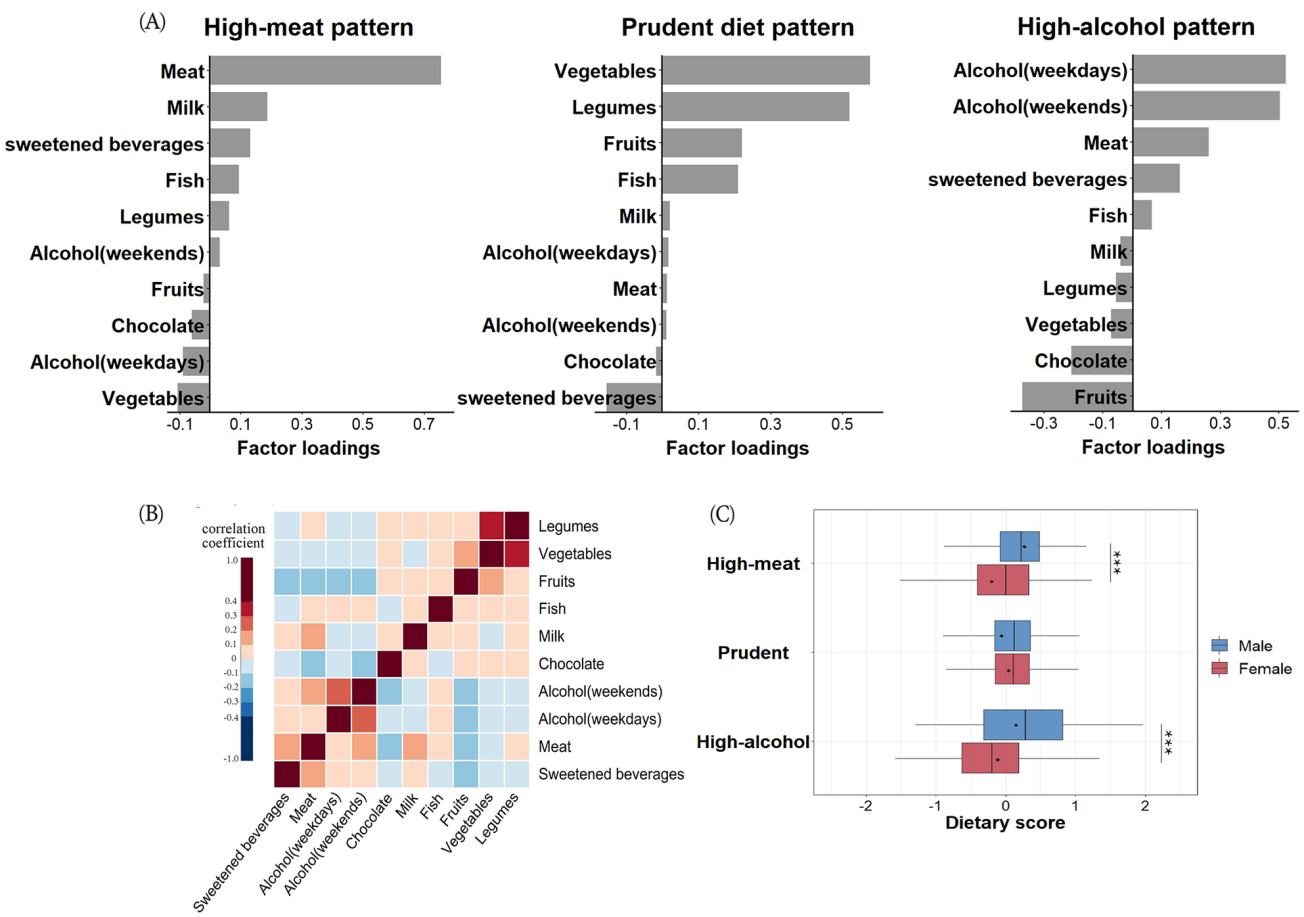
Dietary patterns derived by polychoric correlation analysis. (**A**) Factor loadings for the 10 food groups used in the extraction of the most common dietary patterns among all subjects (n = 482). In high-meat pattern, meat consumption has the highest loading (0.754); In prudent diet pattern, the top four loadings were vegetables (0.575), legumes (0.519), fruit (0.220) and fish (0.209); In high-alcohol consumption, daily (0.522) and weekend (0.503) alcohol consumption weighted the highest. Factor loadings were derived by polychoric correlation with varimax rotation. (**B**) Correlation matrix shows paired correlations of all food items with hierarchical clustering. Red color indicates positive correlations and blue color indicates negative correlations. (**C**) Boxplots show the differences of scores of three dietary patterns between men and women by Wilcoxon test, ***: *P* < 0.001.

The characteristics of dietary patterns obtained a posteriori by polychoric correlation analysis were presented in Fig. 1A,B. Three dietary patterns were detected, together explaining 21.9% of the variation. These were labeled as (1) high-meat pattern, characterized by a high consumption of meat and sweetened beverages, low consumption of vegetables and fruits, (2) prudent diet pattern, mainly characterized by high intake of fish, fruit, legumes and vegetable, low consumption of meat and sugar, and (3) high-alcohol pattern, characterized by higher consumption of alcohol and moderate consumption of meat and sweetened drink. Some differences in food selection also existed between men and women, as displayed in Fig. 1C. Women were less likely to have a high-meat pattern (dietary score − 0.11 vs 0.14, *P* < 0.001) and high-alcohol pattern (dietary score − 0.20 vs 0.26, *P* < 0.001) than men. Women also had a tendency towards higher prudent dietary scores than men but this difference was not significant (dietary score 0.04 vs − 0.05, *P* = 0.133).

### Dietary patterns and circulating inflammatory markers

**Figure 2:**
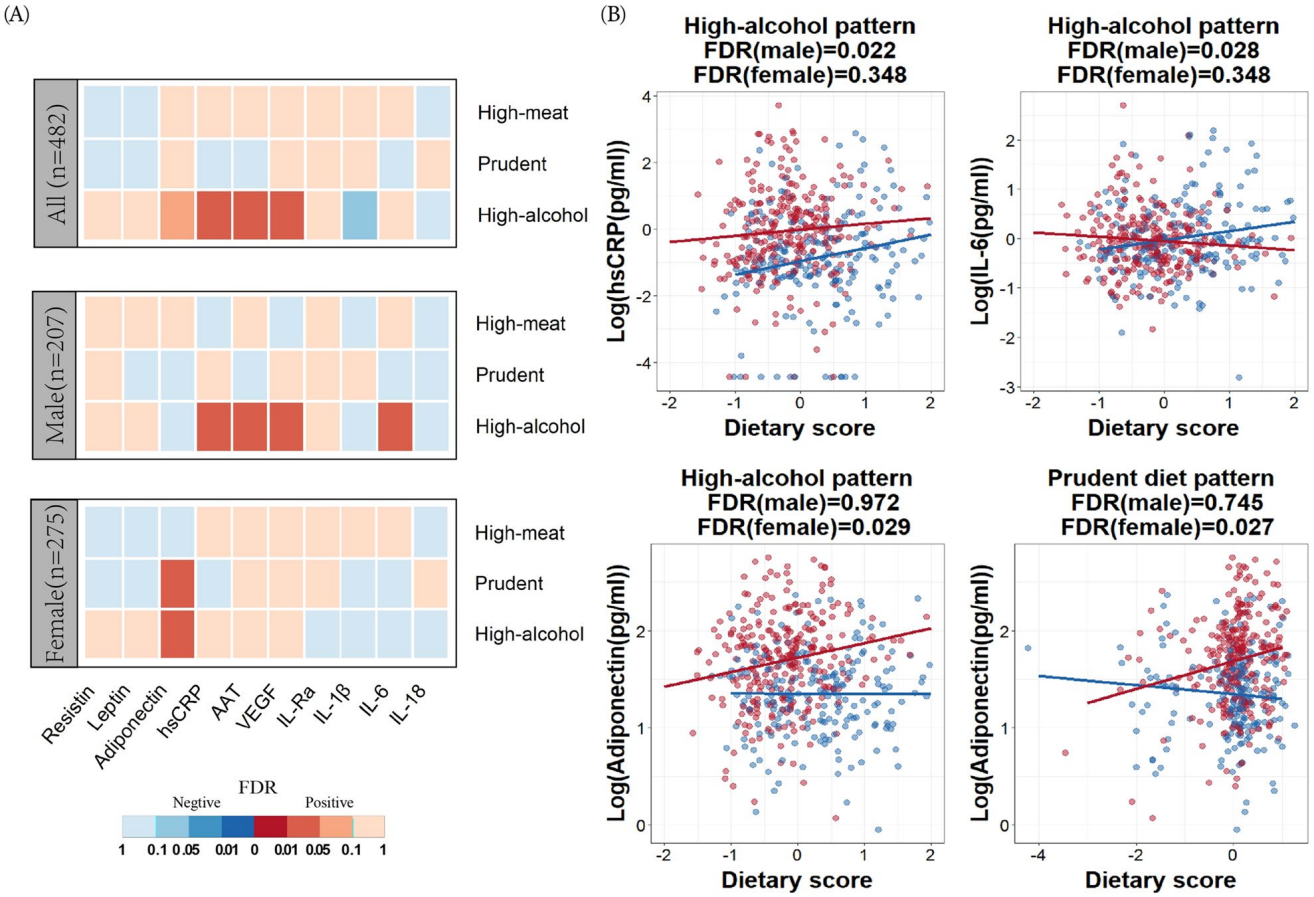
Association between scores of dietary patterns and cytokines concentration. (**A**) The *P* values (FDR corrected per column) of the regression between each dietary pattern and circulating inflammatory cytokines by log transformation. Red in figure legend indicates an FDR with positive correlation and blue indicates FDR with negative correlation. (**B**) Scatterplots show the effect of dietary pattern on cytokines with significant correlation. Blue dots and lines indicate males, red dots and lines indicate females.

Among all participants, after adjustment for additional covariates (sex, age, BMI, smoking, education levels and physical exercise) and other dietary patterns mutually, a positive association between the prudent diet score and the concentration of adiponectin in the circulation of women was observed (FDR *P* = 0.027 in women vs. FDR *P* = 0.745 in men) (see Fig. 2A and corresponding illustration in Fig. 2B). Additionally, the high-alcohol pattern was positively associated with a pro-inflammatory profile (see Fig. 2A). When adjusted for demographics and lifestyle characteristics among all subjects, we observed that high-alcohol diet was significantly associated with hsCRP concentrations (FDR *P* = 0.020), VEGF (FDR *P* = 0.019) and AAT (FDR *P* = 0.034).

When analyzed separately for both men and women, we observed significant positive associations between the high-meat pattern score and serum levels of hsCRP (FDR *P* = 0.022), VEGF (FDR *P* = 0.022), IL-6 (FDR *P* = 0.028) and AAT (FDR *P* = 0.022) in men. In women, we observed a similar trend, though this was not significant after correcting for multiple testing. We also found that the circulating concentrations of adiponectin were positively correlated with a high-alcohol pattern score in women (FDR *P* = 0.029, see Fig. 2A and corresponding illustration in 2B). Interestingly, men did not show this association (FDR *P* = 0.972).

### Mediation by the gut microbial species *Dialister invisus*

**Figure 3:**
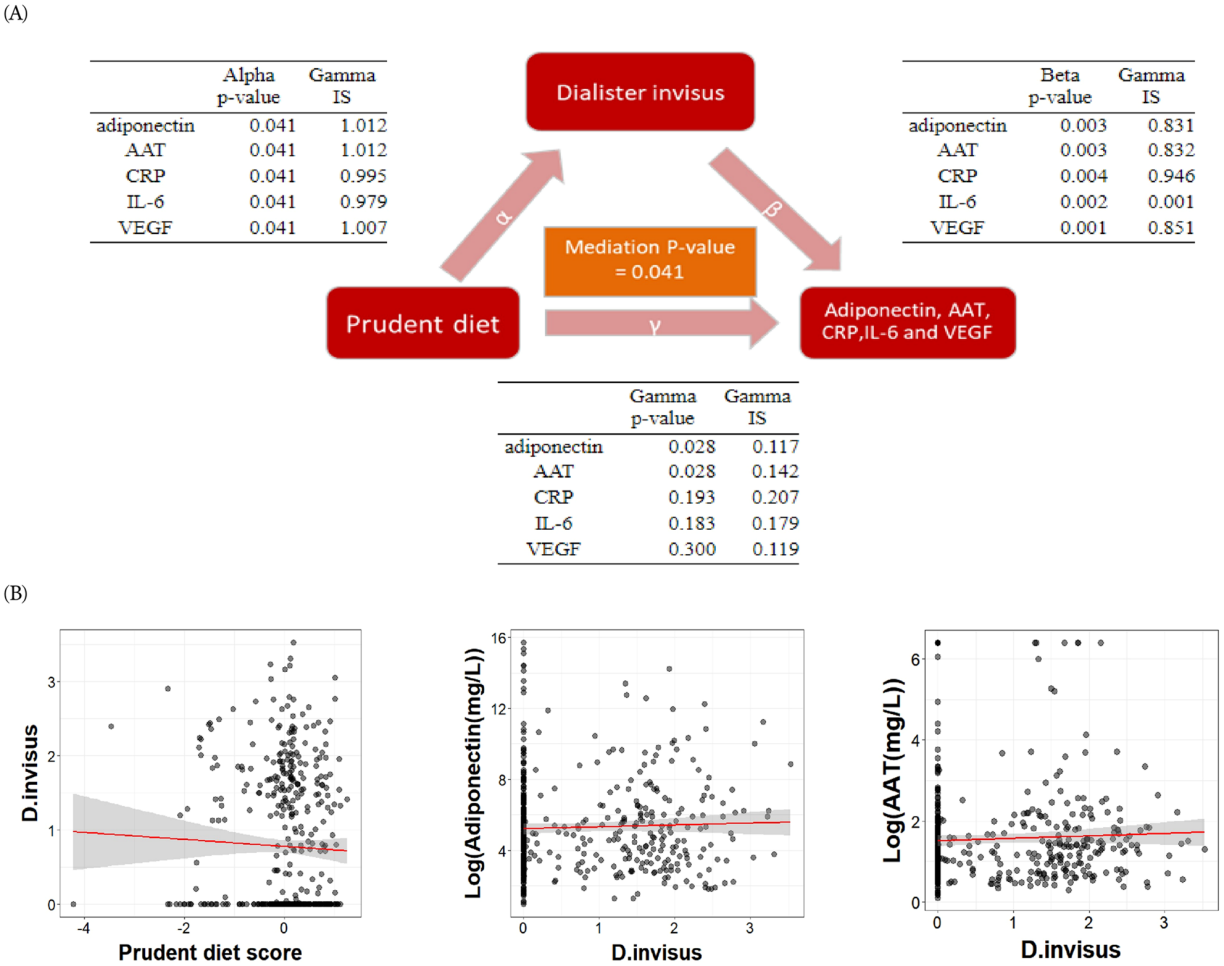
Results of the NPEM method depicted in the mediation framework. (**A**) *Dialister invisus* mediates the relationship between the prudent diet and diponectin, AAT, CRP, IL-6 and VEGF (*P* = 0.041). The alpha, beta and gamma pathways represents the effect of the prudent diet on *Dialister invisus*, the effect of *Dialister invisus* on the immune factors, and the effect of the prudent diet on the immune factors, respectively. FDR corrected p-values of the pathways are shown for each immune factor. IF: information score. (**B**) Scatterplots show the relationship between prudent diet on *D.invisus* and *D.invisus* on immune markers, the unit is 1 out of 100.

The UV algorithm^14^ (which uses abundance values) was used to perform mediation analyses with the three dietary patterns and 75 prevalent species. Five separate mediation analyses were performed, one analysis per immune factor. We found that *D. invisus* was a significant mediator of the relationship between the prudent diet and the immune factors adiponectin, AAT, CRP, IL-6, and VEGF (FDR *P* = 0.041 for each immune factor). Figure 3 shows the significance of the relationships between the prudent diet, *D. invisus*, and the immune factors in the mediation framework.

## Discussion

This study aimed to evaluate potential associations between common dietary patterns of Western-European adults and a set of soluble circulating markers as well as to explore the role of microbiota in the interaction between food and immune system. The positive association between prudent diet and adiponectin and negative association between alcohol consumption with CRP, VEGF and IL-6 has been reported previously^15,16^, but a higher level of AAT with increased alcohol intake has rarely been demonstrated. Besides, we also extended the diet-immune relation by showing that the effect of food on immune factors appears at least partially to be mediated by the GM.

In this cohort, three empirical dietary patters were derived that characterized the subjects’ food consumption. The “high meat” pattern is similar to the high meat diet^17^ and high intakes of vegetables, fruit, legumes and fish have consistently been used to characterize the healthy/prudent dietary pattern in European countries^18,19^. The ‘high-alcohol pattern’ is analogous to similarly named ‘drinker pattern’ in a previous study of other Western populations^20^. Therefore, our extracted dietary patterns are generally consistent with previous studies.

A number of studies have shown the association between the dietary intake of vegetable and fruit, legumes, and fish and increased adiponectin levels^21–23^. The association between higher prudent diet scores and higher serum adiponectin concentrations in our study gives further weight to previous literature. Increased adiponectin concentrations have been reported to be positively associated with insulin sensitization, glucose use, β-oxidation, and cardiovascular protection^24^. The antioxidant properties of vegetable and fruit promote secretion of adiponectin^25^. Eicosapentaenoic acid (EPA) and docosahexaenoic acid (DHA), which are rich in fish, also showed a beneficial effect on increasing plasma adiponectin levels in an animal model^26^. The present study found that prudent diet was significantly associated with higher levels of adiponectin in women, but not in men. A possible explanation is that compliance with prudent diet is higher in women than in men in present study, since prior studies have shown a positive association between plasma levels of adiponectin and adherence to healthy diet in adult men^27^ and women^28^.”

Based on literature, a higher alcohol consumption is expected to be associated with low-grade inflammation, especially among men^29^. In our study, we indeed observed a positive association between a high-alcohol consumption pattern and the concentration of several pro-inflammatory circulating soluble factors such as CRP, IL-6 and VEGF (for men). High concentrations of alcohol promotes the generation of free-radicals in the liver, therefore increasing lipid peroxidation and heightening the level of tissue inflammation^30^. In the study presented here, men had a higher alcohol intake than women and made up 76.2% of population at the highest quintile of high-alcohol pattern. Our results lend support to the prior expectations suggesting that excessive drinking increases risk of multiple chronic diseases^31^. Interestingly, we also observed alcohol intake was positively associated with serum concentration of adiponectin, an anti-inflammatory factor, but only in women. Some cross-sectional studies already revealed a positive association between alcohol consumption and adiponectin^32^, and this association was stronger for women than men^33^. This may result from the activated effect of alcohol on adiponectin-associated gene expression (sirtuin-1/adiponectin-signalling cascade) in fat tissue^34^, and women have a higher percentage of adipose tissue than men^35^. However, a prospective study challenged these results and demonstrated that alcohol consumption was not associated with changes in adiponectin circulating concentrations over time for both sexes^36^. Therefore, the protective effect of moderate alcohol use in terms of adiponectin production requires further exploration. In addition, AAT has not often been reported to be associated with diet. However, AAT has been shown to be similarly elevated as CRP in the acute phase, this was previously found to be the case in autoimmune disease^37^, and can be considered as a mirror of systemic inflammation.

To our knowledge, the ability of *D. invisus* to serve as a mediator between diet and immune parameters has never been shown before. Interestingly, *D. invisus* was first isolated from dental root infection^38^, but also been detected as part of the normal intestinal microbiota^39^. Although this species has not been fully functionally characterized, its end-products acetate and propionate^38^ may explain its mediating effect in the association between prudent diet and lower level pro-inflammatory factors. As it is well known that short-chain fatty acids, such as butyrate, propionate and acetate may promote resolution of intestinal inflammation in animal models^40^ and upregulate adipokine production in type 2 diabetes-derived human adipocytes^41^. *D. invisus* has been implicated in oral infections and had a low abundance in patients with inflammatory bowel disease, which are additional hits that it might have immune-modulatory effects^42,43^.

Adiponectin is the only immune factor that was significantly correlated with the prudent diet when GM was not included in the model (FDR *P* = 0.028 for both immune factors). Further, there was no significant relation between the prudent diet and VEGF, IL-6 and CRP when GM was not taken into account. Therefore, some would argue that *D. invisus* is only a true mediator for adiponectin. However, it is undecided whether an independent correlation needs to be present in order for a mediation effect to be significant^44^. One reason some associations were only significant when a mediator was included in the model is that the direction of relation of the prudent diet with *D. invisus* can be opposite to the relation between *D. invisus* and the immune factors, so that these effects cancel each other out^44^. In this case, there is still a relation between the prudent diet and these factors but this only becomes visible through inclusion of *D. invisus*.

There are some limitations to the study presented here. Firstly, this cross-sectional analysis does not allow establishing causal relationships between dietary factors and immune markers. Second, the food questionnaire was not standardized, which might attenuate the observed effects. Clearly, the finding of mediating effect of bacteria requires further testing in experimental studies to claim or refute causality. In addition, in order to improve power and specificity of the results, mediation methods for the GM should be developed further to support the inclusion of covariates. There are multiple factors besides diet and the GM than have been found to influence circulating immune markers such as sex, age, seasonality, genetics, and BMI^45,46^.

In conclusion, within the subjects of 500FG cohort we identified three dietary patterns. Among these, a prudent dietary pattern was associated with increased adiponectin concentrations. The high-alcohol pattern, characterized by more frequent alcohol consumption, was associated with increased circulating concentrations of pro-inflammatory markers. Our results lend support to the hypothesis that variation in diet is associated with variation in levels of inflammation markers, and further longitudinal studies are needed to confirm the role of inflammatory pathways in chronic diseases. Additionally, our study uncovers a mediating role of *D. invisus* in the relationship between a prudent diet and immune factors in a healthy population. This creates further possibilities to modulate the effect that dietary habits have on immune markers through manipulation of the GM.

## Methods

### Study design and ethics statement

This study is part of the Human Functional Genomics Project (HFGP) (https://hfgp.bbmri.nl/) and uses the 500 Functional Genomics (500FG) cohort, a population-based cohort comprising of 500 healthy Western European adults, which aims to identify the determinants accountable for the variability of immune responses in human body (Data publicly available at: https://hfgp.bbmri.nl/ menu/main/ home). A detailed description of the design and protocol of the 500FG cohort has been described previously^45–47^. Briefly, sampling of 534 healthy participants (237 males and 296 females) of Western European ancestry aged > 18 years was performed at Radboud University Medical Center, Netherlands from August 2013 to December 2014. After blood collection in hospital, participants completed a self-administered questionnaire that contained questions related to demographics, lifestyles, food intake and medical histories (Supplementary Table 1). Primary exclusion criteria in the final analysis was participants who had been treated with medication, and/or had a history of kidney diseases or diabetes mellitus (n = 45) or those whose food questionnaire were not completed (n = 7), leaving 482 eligible individuals for the analysis of diet-circulating markers correlations, and 471 subjects of them for GM analysis.

The study received ethical clearance from Ethical Committee of Radboud University Nijmegen (NL42561.091.12, 2012/550) to ensure subjects protection and adherence to the principles expressed in Declaration of Helsinki. All participants provided written informed consent at study entry.

### Assessment of dietary intake

A semiquantitative food questionnaire was employed to assess usual intake frequency of 10 core foodstuff and beverage groups during the previous year, with an overall response rate of 96.5%. The 4 responses ranged from “never” to “everyday” for the questions on processed meat, fish, fruit, legumes, vegetable and sweeten beverage, while information on consumption of chocolate (bar of 200 g/month), milk (glass of 200 ml/day), alcohol at weekdays(glass/day) and weekends(glass/day) was collected in a form of options with increased amount. Legumes were not included as vegetables^48^, due to the inconsistent classification of legumes as a subcategory of vegetables^49^ (See Supplementary material for more details). All responses were regarded as ordered categorical variables, as described later.

### Lab assessments

#### Circulating markers

Three adipokines (leptin, adiponectin, and resistin) and two acute phase proteins (CRP and alpha-1 antitrypsin (AAT)) were measured with DuoSet enzyme-linked immune- sorbent assay (ELISA) kits following standards from R&D Systems as described previously^45^. Plasma concentrations of IL-1Ra and IL-18 binding protein (IL-18BP) were determined by commercially available Quantikine® kits and protocols (R&D Systems). The serum levels of IL-1β, IL-6, IL-18 and vascular endothelial growth factor (VEGF) were assayed in Simple Plex™ microfluidic cartridges by using Ella instruments (ProteinSimple).

#### Fecal samples and sequencing

Participants were provided with kits and instructions for stool collection at home and storage in the fridge. After delivery to the hospital, stool samples were aliquoted and stored at -80 ℃ until DNA extraction. DNA isolation from stool was done with the use of the AllPrep DNA/RNA Mini Kit (QIAGEN) with the addition of mechanical lysis. DNA concentrations were subsequently measured with the use of the Quant-iT dsDNA Assay Kit and normalized to a concentration of 50 pg/mL. Libraries were prepped for metagenome sequencing on an Illumina HiSeq 2000 platform, sequencing paired-end reads (2*101 base pairs). Please see supplementary materials for the sequencing depth for the microbiome data.

### Statistical approach

#### Relation between dietary patterns and immune markers

The population-level dietary patterns were extracted with exploratory factor analysis, using polychoric correlation analysis with the R package “psych”^50^, a data-driven technique to handle ordered categorical data^51^ (see http://dwoll.de/rexrepos/posts/multFApoly.html for more details). The number of factors retained was based on the output of R function “fa.parallel.poly” by parallel analysis and empirical interpretability of the factors. Factor loadings were derived by varimax rotation and food items were considered to load on a factor dependent on the highest absolute correlation. Dietary score (namely factor score) coefficients were estimated by regression approach and saved for the individual values of the food pattern. The naming of the rotated factors was based on the variables with the strongest contribution and based on prior literature.

Because the concentrations of circulating immune markers were not normally distributed, circulating markers were log-transformed before regression analysis. Values that were below the lower limit of detection were set to the threshold value of the kit. The percentage of missing value is as follows: dietary questionnaire 3.1% and circulating markers 1.5%. Missing values in diet were imputed with the mode (the value that occurs most often) and immune markers were not imputed.

The associations between the derived dietary patterns and the immune biomarkers were evaluated using multiple regression analysis with ‘lm’ function in R language. The regression analysis was conducted separately for each pattern considered, with the continuous dietary score as independent variable and immune characteristics as dependent variables. The potential confounders were chosen on the basis of their associations of the immune markers as indicated in literature: sex, age, smoking status (current smoker, former smoker, passive smoker and never smoker), weekly physical exercise (no exercise, less than twice per week and twice or more exercise/week), body mass index (BMI, calculated as weight/height^2^), educational attainment (primary or secondary, current college training or college). Moreover, we mutually adjusted for other food patterns.

#### Gut microbiota selection

As part of the QC samples with lower than 4 million sequencing reads were removed. Furthermore, forward and reverse reads were combined, filtered based on a minimal read length, and non-bacterial DNA was removed. Taxonomic profiling was done using MetaPhlAn 2.2. For more information on these procedures see^11^. The analysis focused on taxa at species level, encompassing the highest resolution possible in this dataset. Relative abundance was available of 408 microbial species (see Supplementary materials for PCoA plot with bray curtis distances). These abundances were made compositional by dividing the Relative abundance per species by the total read count across all species per sample. To reduce the number of zeros in the abundance data, we selected only the species with a prevalence of ≥ 3% in all samples, with a count detection threshold of 0.01. After this cutoff, we were left with a total of 75 prevalent species. We used this set of species as a starting point for the mediation analyses.

To account for multiple testing between biomarkers and food patterns, an adjusted p-value was calculated with Benjamini–Hochberg procedure^52^ and a threshold of false discovery rate (FDR) < 0.05 was considered as indicating statistical significance. All other statistical analyses were conducted in the R programming language^53^.

#### Mediation analysis

**Figure 4:**
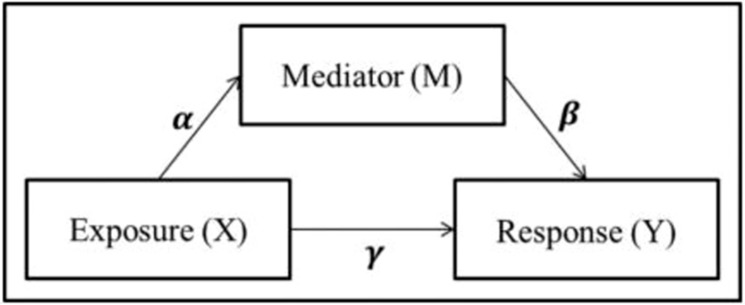
Representation of a mediation model with an exposure (X), mediator (M) and response (Y) and their relationships (α, β, γ).

Mediation analysis was used to investigate whether the identified diet-immune relations are mediated by microbial species. In general, mediation analysis aims to extract the mechanisms by which an exposure (X) impacts the outcome variable (Y) by considering a set of potential variables or mediators which may mediate the effect (M) (Fig. 4).

The characteristics of GM data such as compositionality, high dimensionality and sparsity violate the assumptions made by linear regression. Therefore, non-parametric entropy mediation (NPEM) was employed to perform mediation analysis. The NPEM is based on information theory and uses entropy to calculate the variation and thereby the information that variables contain. Effect is explored by measuring the conditional mutual information between the mediator (M_i) with the exposure set (alpha) and response (beta). The output provided by our function is the mutual information score (which reflects the predictive power of the exposure to the mediator and the mediator to the response)^14^. NPEM analysis was performed in R with a function supplied by Carter et.al^14^. The NPEM algorithm identifies relevant mediators explicitly in the context of noise. Therefore, it relies on the inclusion of the complete dataset rather than for example the selection of specific microbial targets. The NPEM analysis was performed with all selected species and diets at once, but separately for each immune factor, yielding a total of five analyses. Accordingly, the input to the algorithm consisted of: X = three dietary pattern adherence scores, M = log transformed relative abundances of 75 species, and Y = concentration of one of the immune factors. Information score was a score calculated based on information theory and was comparable to coefficient in parametric mediation analysis^14^.

GM: gut microbiome; NPEM: Non-parametric entropy mediation; CRP: C-reactive protein; IL-6: interleukin-6; AAT: Anti-Alpha-1-Antitrypsin; VEGF: vascular endothelial growth factor; QC: quality control.

### Ethics approval and consent to participate

The study received ethical clearance from Ethical Committee of Radboud University Nijmegen (NL42561.091.12, 2012/550) to ensure subjects protection and adherence tot the principles expressed in Declaration of Helsinki. All participants provided written informed consent at study entry.

## Supplementary Information

Supplementary Information 1.

Supplementary Information 2.

Supplementary Information 3.

Supplementary Information 4.

Supplementary Information 5.

Supplementary Information 6.

Supplementary Information 7.

Supplementary Information 8.

Supplementary Information 9.

Supplementary Information 10.

Supplementary Information 11.

## Data Availability

The data collected and/or analyzed in this study are accessible from the corresponding author with reasonable demand.

## Abbreviations

GM: Gut microbiome
NPEM: Non-parametric entropy mediation
CRP: C-reactive protein
IL-6: Interleukin-6
AAT: Anti-alpha-1-antitrypsin
VEGF: Vascular endothelial growth factor
QC: Quality control
