# Supplementary material for: The gut microbiome as mediator between diet and its impact on immune function
Source: Sci Rep. 2022 Mar 25;12:5149. doi: 10.1038/s41598-022-08544-y (PMC8956630; doi:10.1038/s41598-022-08544-y)
Supplement: Supplementary file 1 — Supplementary Information 1. [file 41598_2022_8544_MOESM1_ESM.docx]

Supplemental materials 1: Food questionnaire


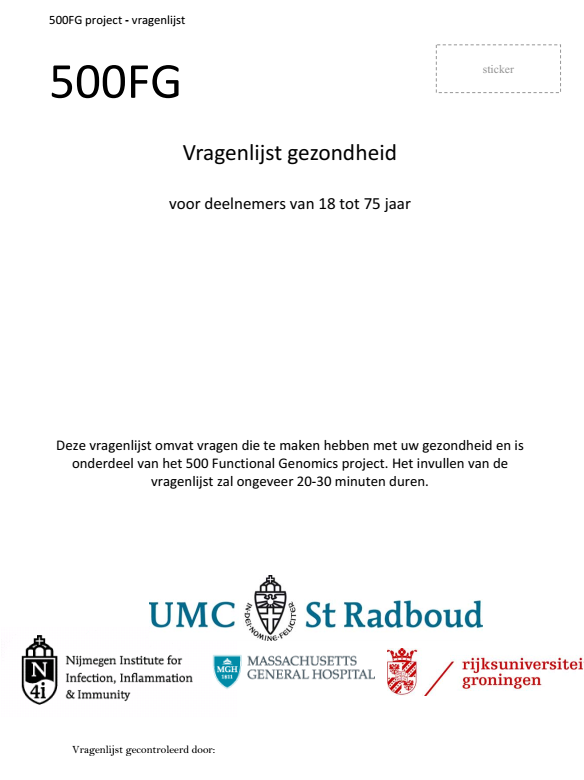


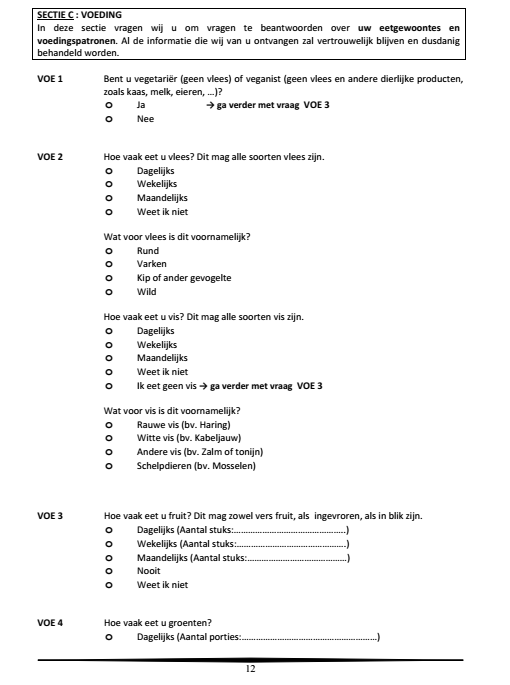


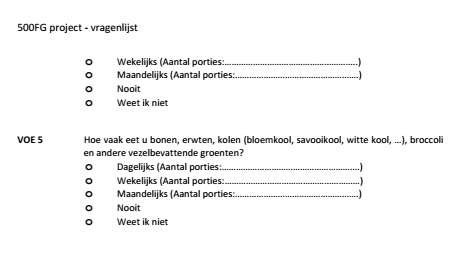


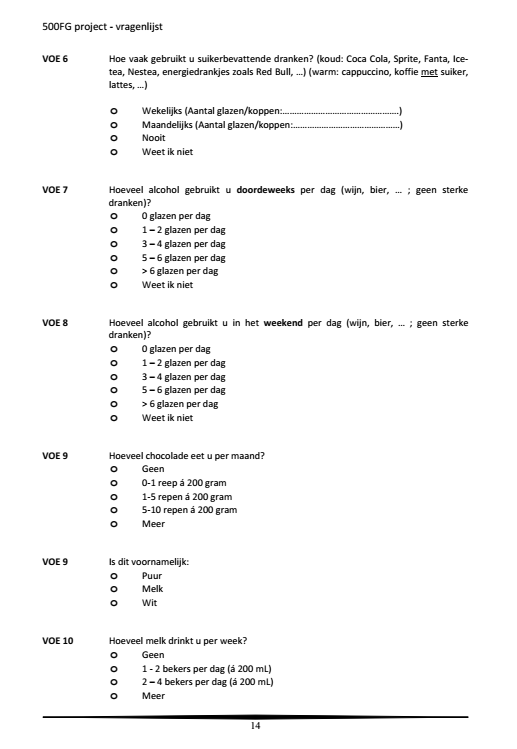


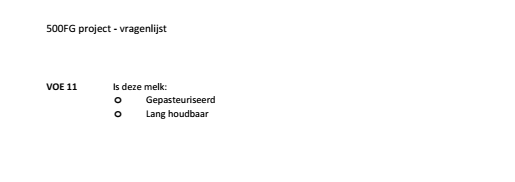


English translation

VOE 1 Are you vegetarian (no meat) or vegan (no meat and other animal products,

such as cheese, milk, eggs, …)?

o Yes → continue with question VOE 3

o No

VOE 2 How often do you eat meat? This can be any type of meat.

o Daily

o Weekly

o Monthly

o I don't know

What kind of meat is this mainly?

o beef

o Pig

o Chicken or other poultry

o Wild

How often do you eat fish? This can be any kind of fish.

o Daily

o Weekly

o Monthly

o I don't know

o I do not eat fish → continue with question VOE 3

What kind of fish is this mainly?

o Raw fish (e.g. herring)

o White fish (e.g. cod)

o Other fish (e.g. salmon or tuna)

o Shellfish (e.g. mussels)

VOE 3 How often do you eat fruit? This can be fresh fruit, frozen or canned.

o Daily (Number of pieces:……………………………………..)

o Weekly (Number of pieces:……………………………………….)

o Monthly (Number of pieces:……………………………………)

o Never

o I don't know

VOE 4 How often do you eat vegetables?

o Daily (Number of servings:…………………………………………………)

o Weekly (Number of servings:…………..)

o Monthly (Number of servings:…………………………………………….)

o Never

o I don't know

VOE 5 How often do you eat beans, peas, cabbages (cauliflower, Savoy cabbage, white cabbage, …), broccoli

and other fiber-containing vegetables?

o Daily (Number of servings:……………….)

o Weekly (Number of servings:………………)

o Monthly (Number of servings:…………………………………………….)

o Never

o I don't know

VOE 6 How often do you consume sugary drinks? (cold: Coca Cola, Sprite, Fanta, Ice‐

tea, Nestea, energy drinks such as Red Bull, …) (hot: cappuccino, coffee with sugar,

lattes,...)

o Weekly (Number of glasses/cups:………………………………………….)

o Monthly (Number of glasses/cups:………………………………………)

o Never

o I don't know

VOE 7 How much alcohol do you consume per day during the week (wine, beer, … ; not strong

drinks)?

o 0 glasses per day

o 1 – 2 glasses per day

o 3 – 4 glasses a day

o 5 – 6 glasses a day

o > 6 glasses a day

o I don't know

VOE 8 How much alcohol do you consume per day at the weekend (wine, beer, … ; not strong

drinks)?

o 0 glasses per day

o 1 – 2 glasses per day

o 3 – 4 glasses a day

o 5 – 6 glasses a day

o > 6 glasses a day

o I don't know

VOE 9 How much chocolate do you eat per month?

o None

o 0‐1 bar of 200 grams

o 1‐5 bars of 200 grams

o 5‐10 bars of 200 grams

o More

VOE 9A Is this mainly:

o Pure

o Milk

o White

VOE 10 How much milk do you drink per week?

o None

o 1 ‐ 2 cups per day (á 200 mL)

o 2 – 4 cups per day (á 200 mL)

o More

VOE 11 Is deze melk:

o Gepasteuriseerd

o Lang houdbaar

| **Supplement table 1**. Characteristics by quintile (Q) of dietary pattern scores by gender. | | | | | | | |
| --- | --- | --- | --- | --- | --- | --- | --- |
|  | | | Age | BMI | Smoking | No Physical activity | College  education |
|  | | | y |  | % | % | % |
| Men | | |  |  |  |  |  |
|  | High-meat | |  |  |  |  |  |
|  |  | Q1 | 28.1 ± 12.5 | 22.6 ± 2.7 | 16.0 | 44.0 | 88.0 |
|  |  | Q3 | 30.0 ± 15.5 | 23.4 ± 3.1 | 16.2 | 23.2 | 81.3 |
|  |  | Q5 | 27.1 ± 10.5 | 23.6 ± 2.9 | 15.6 | 23.5 | 88.4 |
|  |  | P value^a^ | 0.82 | 0.042 | 0.912 | 0.118 | 0.818 |
|  | Prudent diet | |  |  |  |  |  |
|  |  | Q1 | 25.4 ± 9.8 | 23.4 ± 3.0 | 20.0 | 40.0 | 85.4 |
|  |  | Q3 | 33.8 ± 16.8 | 23.3 ± 2.8 | 23.9 | 41.3 | 80.4 |
|  |  | Q5 | 30.9 ± 15.5 | 23.6 ± 2.8 | 22.2 | 22.2 | 81.0 |
|  |  | P value^a^ | 0.001 | 0.865 | 0.072 | 0.022 | 0.428 |
|  | High-alcohol | |  |  |  |  |  |
|  |  | Q1 | 34.9 ± 17.0 | 23.4 ± 3.6 | 10.3 | 20.6 | 65.5 |
|  |  | Q3 | 32.5 ± 15.8 | 23.1 ± 3.2 | 8.82 | 23.5 | 91.1 |
|  |  | Q5 | 25.5 ± 10.5 | 23.3 ± 2.0 | 27.9 | 32.3 | 89.8 |
|  |  | P value^a^ | 0.005 | 0.982 | 0.001 | 0.064 | 0.011 |
| Women | | |  |  |  |  |  |
|  | High-meat | |  |  |  |  |  |
|  |  | Q1 | 27.4 ± 13.1 | 22.2 ± 2.7 | 5.71 | 36.1 | 84.7 |
|  |  | Q3 | 25.4 ± 10.3 | 21.9 ± 2.0 | 13.2 | 33.9 | 90.5 |
|  |  | Q5 | 28.0 ± 11.8 | 22.9 ± 2.5 | 11.1 | 42.2 | 86.6 |
|  |  | P value^a^ | 0.543 | 0.203 | 0.794 | 0.794 | 0.165 |
|  | Prudent diet | |  |  |  |  |  |
|  |  | Q1 | 25.6 ±11.2 | 22.4 ± 3.0 | 19.0 | 35.7 | 80.9 |
|  |  | Q3 | 24.7 ±10.2 | 21.9 ± 2.2 | 6.12 | 42.0 | 92.0 |
|  |  | Q5 | 29.8 ±14.2 | 22.5 ± 2.5 | 6.66 | 21.6 | 91.6 |
|  |  | P value^a^ | 0.073 | 0.534 | 0.097 | 0.015 | 0.311 |
|  | High-alcohol | |  |  |  |  |  |
|  |  | Q1 | 27.4 ± 11.9 | 23.4 ± 3.6 | 8.82 | 39.7 | 89.7 |
|  |  | Q3 | 27.5 ±12.5 | 23.1 ± 3.2 | 13.1 | 24.1 | 85.4 |
|  |  | Q5 | 22.0 ±3.3 | 23.3 ± 2.0 | 14.2 | 46.4 | 92.8 |
|  |  | P value^a^ | 0.123 | 0.925 | 0.061 | 0.061 | 0.412 |
| ^a^ P values are based on ANOVA for continuous variables and chi-square tests for categorical variables. | | | | | | | |
