# Supplementary figures and images for: The gut microbiome as mediator between diet and its impact on immune function
Source: Sci Rep. 2022 Mar 25;12:5149. doi: 10.1038/s41598-022-08544-y (PMC8956630; doi:10.1038/s41598-022-08544-y)

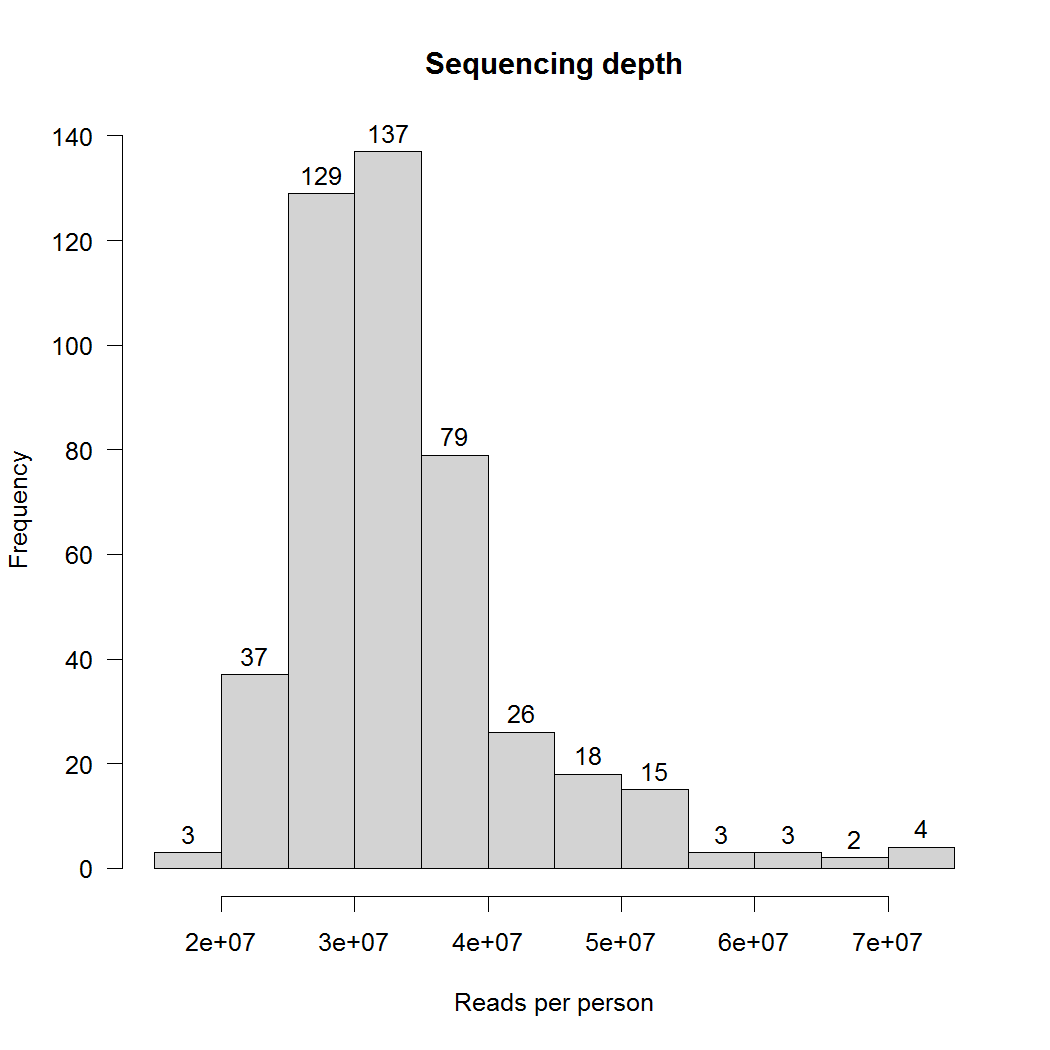

Supplement: Supplementary file 7 — Supplementary Information 7. [file 41598_2022_8544_MOESM7_ESM.jpg]

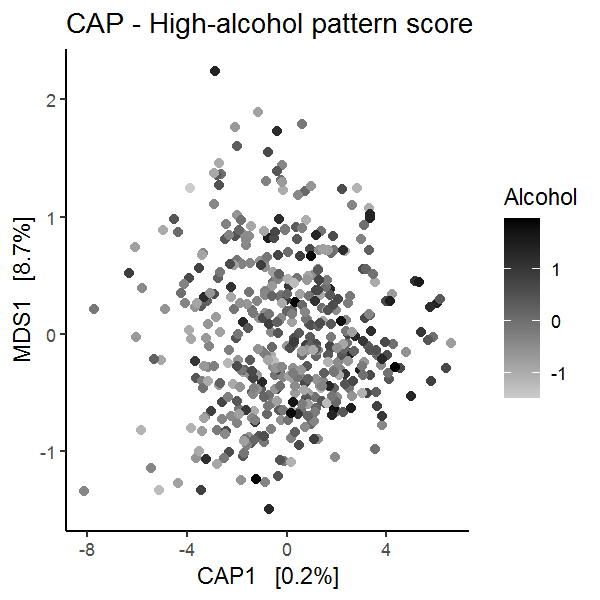

Supplement: Supplementary file 9 — Supplementary Information 9. [file 41598_2022_8544_MOESM9_ESM.png]

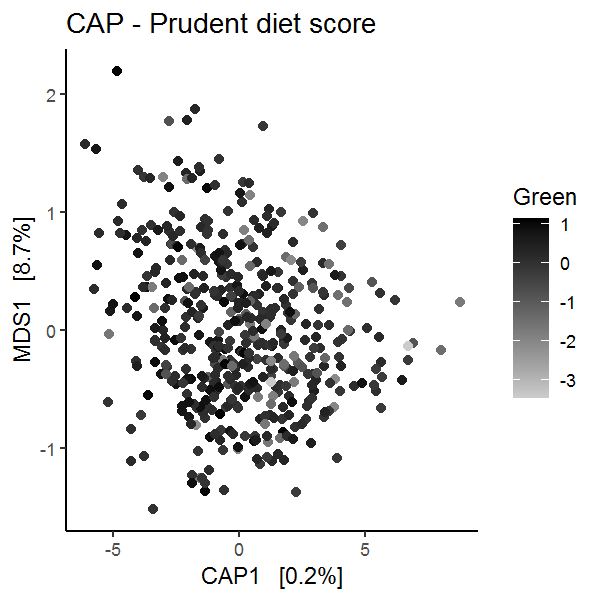

Supplement: Supplementary file 10 — Supplementary Information 10. [file 41598_2022_8544_MOESM10_ESM.png]

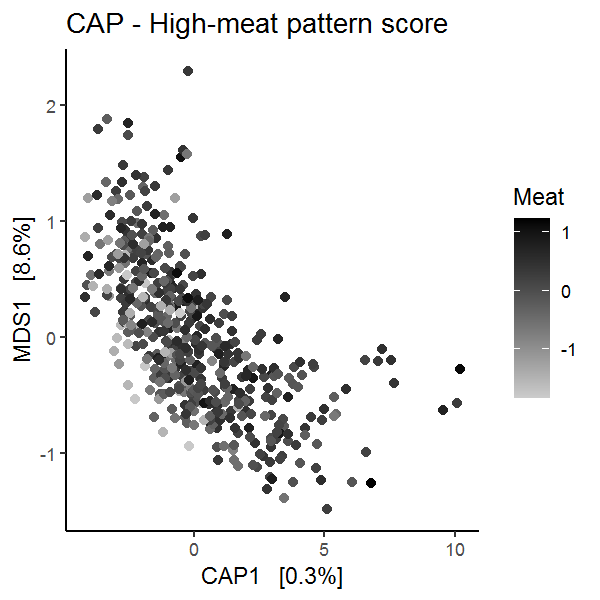

Supplement: Supplementary file 11 — Supplementary Information 11. [file 41598_2022_8544_MOESM11_ESM.png]
